# Editorial: Human-Computer Interaction Serious Games as behavioral change moderators

**DOI:** 10.3389/fpsyg.2022.1115366

**Published:** 2022-12-21

**Authors:** Sofia Balula Dias, José Alves Diniz, Leontios J. Hadjileontiadis, Herbert F. Jelinek

**Affiliations:** ^1^Interdisciplinary Centre for the Study of Human Performance (CIPER), Faculdade de Motricidade Humana, Universidade de Lisboa, Estrada da Costa, Lisbon, Portugal; ^2^Department of Biomedical Engineering, Healthcare Engineering Innovation Center (HEIC), Khalifa University of Science and Technology, Abu Dhabi, United Arab Emirates; ^3^Department of Electrical and Computer Engineering, Aristotle University of Thessaloniki, Thessaloniki, Greece

**Keywords:** Human-Computer Interaction, Serious Games, behavior change mediators, in-game metrics, machine learning

## Introduction

Human-Computer Interaction Serious Games (HCI-SGs) set a novel domain in understanding player motivations in gaming, behavioral implications of game play, game adaptation to player preferences and need for increased engaging experiences. When the latter relate to health status, HCI-SGs can become a part of daily life as assistive health status monitoring and enhancement systems (Dias et al., 2021). A combined view of both game-based learning (Prensky, 2001) and game-based assessment (Ifenthaler et al., 2012) principles can contribute to improvement of game design by applying interdisciplinary approaches and methodologies that allow examination/classification of cognitive, physical, emotional, and motivational processes during gameplay. Generated from the interaction of users of HCI-SGs, in-game metrics can potentially be used as digital biomarkers to predict a player's state of physical and psychological health and wellbeing as well as provide a gaming environment for improvement (Petsani et al., 2022).

As Guest Editors for the Frontiers in Psychology (Human-Media Interaction section), we are delighted to announce that eight papers were accepted after a peer review process that included contributions of more than 20 high-quality international external reviewers and guest editors with expertise in the field of Human-Media Interaction. The accepted manuscripts highlight contributions and advances in the current literature, setting future avenues of research within the HCI-SGs domain, as presented in the next section.

## Contributions

**Figure 1:**
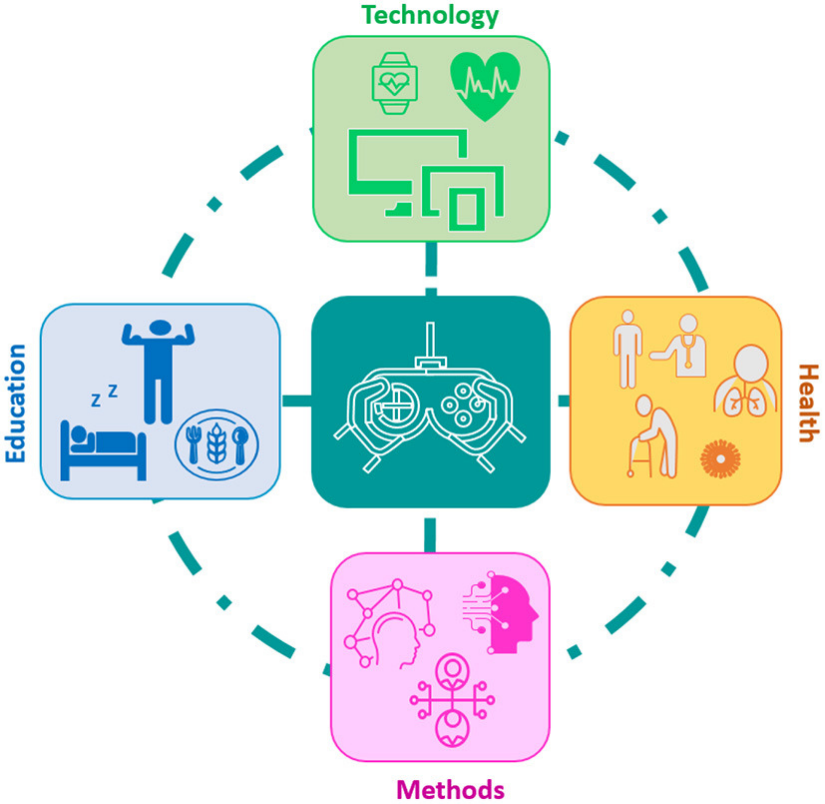
Application areas of Human-Computer Interaction Serious Games, ranging from educational games to games for health and behavior change, along with biosensing technology and computational methods.

The current collection of articles provides validated research, including results from experiments, European projects and real-life applications, based on theories and game applications, in the specific field of HCI-SGs toward behavioral change, covering areas like psychology, (open) education, gamification, rehabilitation, wellbeing, physical and mental health, modeling, machine learning, mobile technology, disability, design process, adaptation, personalization, HCI, co-creation, and emotional intelligence (see Figure 1).

Huang and Chen propose a game-based situational learning system as an anti-drug experiment with 53 junior middle school students (aged 13–15), to explore whether different personality traits result in different anti-drug responses. The authors collected 95 sets of codes (a total of 20,550 behavioral codes) from students during the period of using the serious game-based situational learning system. The codes allowed the authors to extrapolate a behavioral transition diagram *via* Lag Sequential Analysis (LSA) using Generalized Sequential Querier (GSEQ 5.1) software. Different behavioral patterns (such as differentiation, acceptance, effective and ineffective responses and solution seeking, and failure to refuse) were identified, providing important guidelines for designing adaptive anti-drug education programs.

Fernández-Avilés et al. applied results from the 82-item Motivational Trait Questionnaire (MTQ-long version) and the 3D Motivational Traits Game-based Objective Test (MT-GOT) to evaluate motivational traits. To assess the validity of the results of the measured motivational traits using the objective behavioral test 31 participants (with an average of 40 games per participant), whose motivational traits were evaluated and compared using these two methods, were recruited. The results from the statistical analysis suggest that the objective MT-GOT game can be a useful application to assess motivational traits as an alternative to the self-report MTQ questionnaire.

Padilla-Zea et al. present the “Catch the Open!,” a 2D virtual web-based game, targeting Educators who intend to learn or have more knowledge about Open Education (OE) and Open Educational Practices (OEP) within a particular Higher Education Institution (HEI). The educational content integrated in the Catch the Open! Game was based on the OE framework, as an extension of an Erasmus+ OpenGame project. Involving 153 Educators from six HEIs, several tests and pilot studies were performed, both internally (in the consortium) and with potential end-users. Overall, the results show that the “Catch the Open!” game helped in learning more about OE and the gamified web-based interactive application was mostly rated to be useful for its purpose.

Using the Activity Theory (AT) approach, Tlili et al. conducted a systematic review to critically explore and better understand the effects of game-based learning applications for learners with disabilities. Content analysis from 96 empirical studies showed important information related to each AT construct. The authors identified relevant gaps in this field, suggesting, that general and domain-specific recommendations/challenges to support practitioners that intend to use game-based learning for learners with disabilities.

Menychtas et al. introduces a gamified design process for a mobile application (MediLudus) targeting patients in rehabilitation and aiming to maintain and improve their mental and physical health. The authors critically discuss key performance indicators (e.g., engagement, functionality, interaction) that are important to consider during the early evaluation phase of the application in terms of patient progress, including in particular motivation, interest, and degree of adherence to exercise programs.

Theodoropoulos and Lepouras explore the effects of different high school programming environments, by developing games considering gender and personality characteristics. Three groups were considered in this study according to the use of Scratch, App Inventor and Alice 3D, in order to find potential biases based on gender, learning perception, usage and student personalities in the three experimental conditions. Data were collected using pre-/post-activity questionnaires, involving 163 students (aged 14–15 years). The authors found that all programming settings reveal different gender-, personality-, and emotion-related effects on students.

An interesting experimental study designed to explore the effectiveness of the educational “RUEU?” game, and to support higher education students in understanding the key socio-political issues regarding European identity was performed by Jimoyiannis et al.. Data from 92 Greek university students' attitudes to European identity, before and after playing the “RUEU?” game, were collected. The results show that both instructional interventions were effective to deliver academic content, but also to promote students' reflection at an attitudinal and emotional level.

Mahboobeh et al. address important insights, regarding the use of ICT-based tools for capturing the status of Parkinson's disease (PD) patients. Data derived from 27 PD patients at Stage 1 and 3 were analyzed regarding their interaction with the Personalized Serious Game Suite (PGS) and intelligent Motor Assessment Tests (iMAT) gamified environments. Five feature vector (FV) scenarios were fed into machine learning classifiers [i.e., K-Nearest Neighbor (KNN), Support Vector Machines (SVM), and Random Forest (RF)], in order to infer the stage of each PD patient. A Leave-One-Out Cross-Validation (LOOCV) method was adopted, and the experimental results revealed that a high (> 90%) classification accuracy can be achieved from both data sources (PGS/iMAT), showing the effectiveness of PGS/iMAT to efficiently reflect the motor skill status of PD patients. This further potentiates PGS/iMAT enhancement with machine learning techniques to infer the stage of PD patients.

## Summary

This Research Topic clearly shows that HCI-SGs can be transferable and applicable to several fields, such as education, rehabilitation, and training, as they can potentiate the (re)education and improve/sustain heath status of the end-users. The eight papers presented here are based on well-established theories/perspectives (e.g., Activity theory, Game-based learning, Game-based assessment, User-centered approach), showing that HCI-SGs can be specially designed to better support player's active learning, engagement and critical thinking within the game. However, in order to develop new applications, more innovative HCI tools and corresponding methodologies are required in the field. For example, the integration of artificial intelligence within the game co-design/development, including adaptation algorithms, has the capability to change/re-adapt the HCI-SGs at different levels of interaction.

Finally, it is our hope that the scientific community and industry can benefit from the theoretical and practical contributions of this Research Topic, boosting further the field of HCI-SGs and emerging related-areas.

## Author contributions

All authors listed have made a substantial, direct, and intellectual contribution to the work and approved it for publication.
